# Isolation and Characterization of a Novel *Escherichia* Bacteriophage with Potential to Control Multidrug-Resistant Avian Pathogenic *Escherichia coli* and Biofilms

**DOI:** 10.3390/antibiotics13111083

**Published:** 2024-11-13

**Authors:** Phitchayapak Wintachai, Fahsai Thaion, Martha R. J. Clokie, Thotsapol Thomrongsuwannakij

**Affiliations:** 1 Bacteriophage Laboratory, Walailak University, Thasala, Nakhon Si Thammarat 80161, Thailand; thaionfahsai@gmail.com; 2 School of Science, Walailak University, Thasala, Nakhon Si Thammarat 80161, Thailand; 3 Functional Materials and Nanotechnology Center of Excellence, Walailak University, Thasala, Nakhon Si Thammarat 80161, Thailand; 4 Department of Genetics and Genome Biology, University of Leicester, Leicester LE1 7RH, UK; mrjc1@leicester.ac.uk; 5 Akkhraratchakumari Veterinary College, Walailak University, Nakhon Si Thammarat 80161, Thailand; thotsapol.th@wu.ac.th; 6 Centre for One Health, Walailak University, Nakhon Si Thammarat 80161, Thailand

**Keywords:** antibacterial activity, avian pathogenic *Escherichia coli*, bacteriophage, biofilms, phage therapy

## Abstract

**Background/Objectives:** Avian pathogenic *Escherichia coli* (APEC) infection is a significant problem for the global chicken industry, as it decreases animal welfare and is associated with substantial economic losses. Traditionally, APEC infections have been controlled through the use of antibiotics, which has led to an increased prevalence of antibiotic-resistant *E. coli*. Therefore, developing alternative treatments for APEC infection is crucial. **Methods:** In this study, an *Escherichia* phage specific to multidrug-resistant (MDR) APEC, designated as phage vB_EcoP_PW8 (phage vECPW8), was isolated. The morphology, phage adsorption to host cells, one-step growth curve, thermal stability, pH stability, whole-genome sequencing, antibacterial ability, and antibiofilm efficacy of phage vECPW8 were evaluated. **Results:** The results demonstrated that phage vECPW8 has a *Podoviridae* morphology and is effective at lysing bacteria. Phage vECPW8 exhibited a high absorption rate to bacterial cells (more than 85% within 10 min) and had a latent period of 20 min, with a burst size of 143 plaque-forming units per cell. Additionally, phage vECPW8 showed good temperature and pH stability. The phage displayed strong antibacterial activity in vitro, and its efficacy in controlling bacteria was confirmed through scanning electron microscopy. Whole-genome sequencing revealed that the phage has a linear genome with 69,579 base pairs. The genome analysis supported the safety of the phage, as no toxin, virulence, or resistance-related genes were detected. Phage vECPW8 was identified as a novel lytic phage in the *Gamaleyavirus* genus and *Schitoviridae* family. The phage also demonstrated antibiofilm efficacy by reducing and preventing biofilm formation, as evidenced by biofilm biomass and bacterial cell viability measurements. **Conclusions:** These results indicate that phage vECPW8 is a promising candidate for the effective treatment of MDR APEC infections in poultry.

## 1. Introduction

*Escherichia coli*, a member of the *Enterobacteriaceae* family, is divided into commensal and pathogenic types. Pathogenic *E. coli* is classified into intestinal pathogenic *E. coli* (IPEC) and extra-intestinal pathogenic *E. coli* (ExPEC) [1]. Among ExPEC strains, avian pathogenic *E. coli* (APEC) is notable for causing both local and systemic infections in poultry [2]. APEC can infect chickens of all ages, as well as ducks, turkeys, and other bird species. APEC causes colibacillosis, which has a significant economic impact on the global poultry industry [3]. The infections are characterized by perihepatitis, airsacculitis, cellulitis, coligranuloma, pericarditis, egg peritonitis, omphalitis, osteomyelitis/arthritis, perihepatitis, salpingitis, and life-threatening septicemia. 

A previous study showed that APEC isolates share significant genetic similarities with human ExPEC strains, indicating a close relationship with strains that cause infections in both humans and animals [4]. Additionally, APEC may serve as a reservoir for virulent and antibiotic-resistant genes that can be transferred to other ExPEC strains [5]. Both APEC and human ExPEC exhibit the same virulence factors and pathogenic mechanisms [6], highlighting the potential for zoonotic transmission. The virulence and resistance genes can be transferred to humans through direct contact between animals and humans, food animals, and environmental factors [7,8]. Consequently, the zoonotic potential of APEC has raised concerns [9]. Moreover, multidrug-resistant (MDR) APECs have been detected worldwide, with increasing resistance to different antibiotic classes such as aminoglycosides, doxycycline, fluoroquinolones, macrolides, penicillins, and tetracyclines [10,11]. Recent research has also reported that APEC demonstrates resistance to nearly all classes of antibiotics, including carbapenems, which are known for their broad spectrum of activity and effectiveness [12]. Antibiotic-resistant APEC can lead to treatment failure and subsequent economic losses, and also serves as a source of resistant bacteria and genes, posing a significant risk to human health. Thus, alternative treatments for APEC control are of great interest.

A promising alternative method to control bacterial infection is the use of bacteriophages or phages. Phages can kill specific bacteria without affecting non-target bacteria or normal microbiota. Many studies have reported the isolation and characterization of phages specific to APEC. Numerous phages have demonstrated high efficacy in controlling APEC bacterial cells, with a few also showing potential to control biofilms, which is significant as much infection occurs in this form. For example, *Escherichia* phage PEC9, a member of the *Tequatrovirus* genus in the *Straboviridae* family, can lyse APEC and protect mice against APEC infections [13]. *Escherichia* phages SKA49 and SKA64 have shown antibacterial activity against MDR APEC strains [14]. *Escherichia* phage AG- MK-2022 Basu exhibited high bactericidal activity against APEC strains [15]. Phage O78 can significantly reduce biofilms [16]. Additionally, 18 phages specific to APEC, belonging to various genera, were isolated and evaluated as a phage cocktail, demonstrating good efficacy in inhibiting APEC growth [17]. 

Although many studies on phages specific to APEC have been reported, no commercial phage products targeting Thai APEC isolates have been developed for use in broiler and chicken farms. This study addresses this gap by demonstrating the potential of a novel phage as an effective antibacterial and antibiofilm agent, suitable for use in farms and environments across Thailand. The objectives of this study are to isolate and characterize an *Escherichia* phage specific to MDR APEC and to assess its antibacterial and antibiofilm activities. 

## 2. Results

### 2.1. Phage Isolation and Virion Morphology

**Figure 1:**
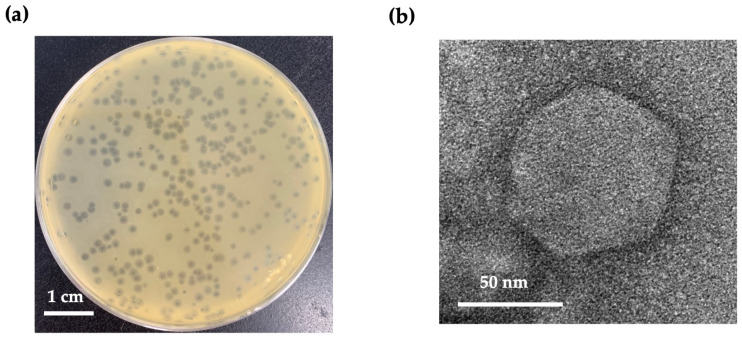
Plaque and virion morphology of *Escherichia* phage vECPW8; (**a**) plaque formation by *Escherichia* phage vECPW8 on a lawn of MDR APEC PW005; (**b**) electron micrograph image of phage vECPW8. The phage was observed at a magnification of 120,000×.

An *Escherichia* phage, vECPW8, was isolated from an environmental water sample. The phage formed small plaques with a halo on a bacterial lawn (1–2 mm in diameter), indicating successful bacterial lysis (Figure 1a). Transmission electron microscopy (TEM) revealed the morphology of the phage, showing an icosahedral head with a diameter of 83.33 (±4.72) nm and a short, non-contractile tail (Figure 1b). Based on these morphological characteristics, the phage was classified within the *Podoviridae* family and designated as *Escherichia* phage vB_EcoP_PW8 (phage vECPW8).

### 2.2. Host Range and Efficiency of Plating (EOP) of Phage

The host range of *Escherichia* phage vECPW8 was evaluated using a spot test assay on 20 MDR APEC isolates obtained from commercial broilers and native chickens [17]. The results showed that *Escherichia* phage vECPW8 could lyse 55% (11/20) of the APEC isolates. The susceptible APEC isolates were then analyzed for EOP. Phage vECPW8 exhibited high activity against four isolates, moderate activity against five isolates, and low activity against two isolates (Table 1). 

### 2.3. Adsorption of Phage

**Figure 2:**
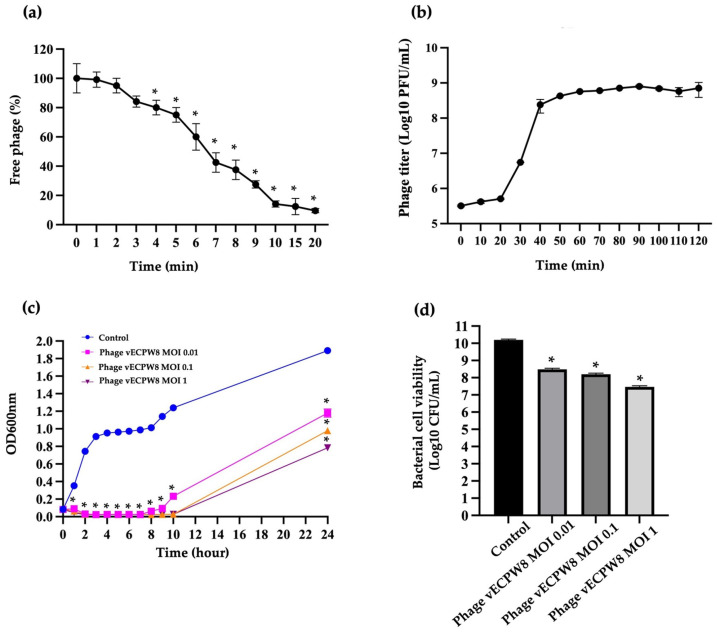
Biological characterization of *Escherichia* phage vECPW8: (**a**) the adsorption rate of phage vECPW8 to host bacteria; (**b**) one-step growth curve of phage vECPW8 on host bacteria; (**c**) bacteriolytic activities of phage vECPW8 on host bacteria; (**d**) the effect of phage vECPW8 treatment on bacterial cell viability at 24 h. Bars represent the standard error of the mean (SEM), and asterisks indicate significant differences (* *p* ≤ 0.05).

Phage vECPW8 demonstrated an adsorption rate of 85.83% into APEC bacterial cells within the first 10 min (Figure 2a). The adsorption efficacy continued to increase, reaching 90.42% at 20 min post-infection. The adsorption rate constant (*k*) for phage vECPW8 was 1.17 × 10^−8^ mL/min. 

### 2.4. One-Step Growth Curve Analysis

A one-step growth experiment was conducted to determine the latent period and the burst size of phage vECPW8. The latent period, defined as the time from phage adsorption to release, was 20 min (Figure 2b). The phage titer increased continuously, reaching a plateau at 60 min. The mean burst size was calculated to be 142.67 plaque-forming units (PFU) per cell. 

### 2.5. Influence of Phage on Lytic Activity 

The inhibitory effect of phage vECPW8 on bacterial growth was assessed by measuring the optical density at 600 nm (OD600). The absorbance of untreated APEC (control) continuously increased over time (Figure 2c). Phage vECPW8 at multiplicities of infection (MOIs) of 0.1 and 1 completely inhibited APEC growth within 1 h, while complete inhibition occurred within 2 h at an MOI of 0.01. Ten hours post-incubation, bacterial growth was consistently suppressed by phage vECPW8 at MOIs of 0.1 and 1. However, bacterial regrowth was observed 8 h after treatment with phage vECPW8 at an MOI of 0.01. After 24 h of incubation, the regrowth of APEC treated with phage vECPW8 at MOIs of 0.01, 0.1, and 1 was detected. The growth was lower than that of the control.

To further assess the impact of phage vECPW8 treatment on bacterial cell viability over 24 h, a significant decrease in cell viability was observed, with a reduction of approximately 1.9 to 2.9 log units following incubation at MOIs of 0.01 to 1, respectively (Figure 2d).

### 2.6. Electron Microscopy of APEC Cells Exposed to Phage 

**Figure 3:**
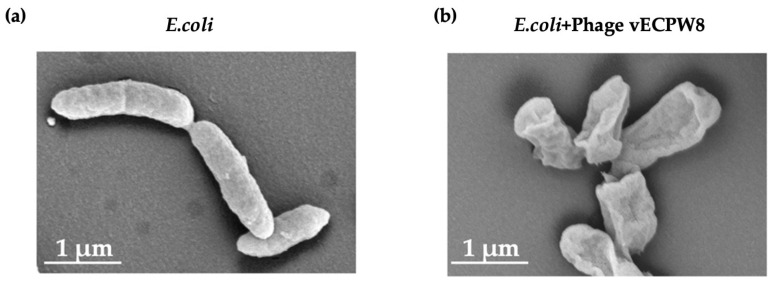
Scanning electron microscopy analysis of APEC infected with *Escherichia* phage vECPW8. (**a**) Untreated bacterial morphology; (**b**) APEC infected with phage vECPW8.

The changes in bacterial cell morphology after phage treatment were investigated using a scanning electron microscope (SEM). The morphology of APEC bacterial cells post-phage treatment was evaluated and compared to untreated APEC bacterial cells, which served as controls. The untreated bacterial cells exhibited a rod shape with smooth and intact cell membranes (Figure 3a). In contrast, all bacterial cells treated with phage vECPW8 showed ruptured membranes. Shrinkage and pore formation were observed in these treated cells, likely leading to cell lysis (Figure 3b). 

### 2.7. Stability of Phage Under Various Temperatures, pH Values, and Ultraviolet Type C (UVC) Radiation 

**Figure 4:**
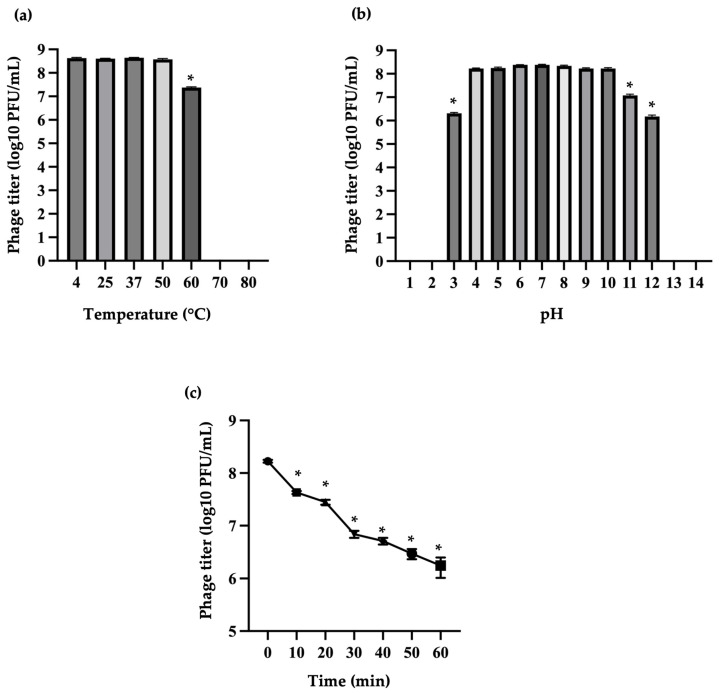
Stability of *Escherichia* phage vECPW8 under various conditions. This study examined the stability of *Escherichia* phage vECPW8 under different conditions, including (**a**) the effects of various temperatures, (**b**) different pH values, and (**c**) exposure to UVC radiation on the survival of the phage. Bars represent the standard error of the mean (SEM) and asterisks indicate significant differences (* *p* ≤ 0.05).

The stability of phage vECPW8 was assessed after a 1 h incubation period under various thermal and pH conditions. Phage vECPW8 remained stable at temperatures between 4 and 50 °C; however, a significant reduction in viability was observed at 60 °C, and no viability was detected at 70 or 80 °C (Figure 4a). Regarding pH stability, phage vECPW8 maintained stability between pH 4 and 10, but stability significantly decreased at pH levels 3, 11, and 12. No viability was observed at extreme pH levels of 1, 2, 13, or 14 (Figure 4b).

The stability of phage vECPW8 was also assessed under UVC radiation. Phage viability was reduced by 74.6% within 10 min (Figure 4c). After that, phage viability continued to decrease, but it could still be detected 60 min after exposure to UVC radiation.

### 2.8. Whole-Genome Sequencing and Bioinformatics Analysis

**Figure 5:**
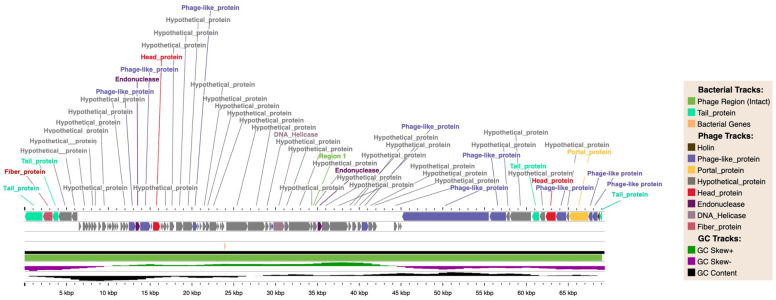
Genome map of *Escherichia* phage vECPW8. The genome map was constructed using the PHASTEST web server. Different colors on the map represent various functional categories.

The genome of phage vECPW8 was sequenced using the Illumina platform. The sequences were de novo assembled into a single contig and annotated. The results revealed that phage vECPW8 has a linear DNA genome, 69,357 base pairs in length, with a G+C content of 42.80%. A genome map was constructed (Figure 5), and the phage was annotated using the PHASTEST web server (Supplementary Table S1), which identified 79 coding sequences (CDSs). These included four CDSs related to the tail protein group: a putative tail spike protein, two putative tail proteins, and a putative tail length tape-measure protein. One CDS encodes for a putative tail fiber protein. Two CDSs in the head protein group are the putative capsid decorating protein and the major coat protein. A further ten CDSs appear to have homologues with phage-encoded proteins, including RNA polymerase RNAP1, RNA polymerase RNAP2, dCTP deaminase, Phi92_gp206, single-stranded DNA binding protein, putative virion RNA polymerase, putative structural proteins, tape-measure protein, putative Rz/Rzl spanin protein, and acetylmuramidase. In terms of putative proteins involved in transcription, there is also one CDS, which is a DNA helicase and phage-associated homing endonuclease. Portal protein and putative holin were detected as CDSs in the portal protein and holin groups, respectively. Moreover, 58 CDSs were classified as 58 hypothetical phage proteins. The depolymerase domain was notably identified on CDS number 1, spanning from DNA base position 2 to 2135. 

Annotation of the phage genome was also performed using the PhageScope program. In this analysis, the phage genome appeared to encode 80 CDSs, including 5 genes related to infection, 8 genes related to assembly, 8 genes related to phage replication, 6 lysis genes, 2 regulatory genes, 1 immune gene, and 36 hypothetical proteins. No integration genes, antimicrobial-resistant genes, virulence genes, or tRNA were detected. According to the PhageScope results, phage vECPW8 belongs to the *Caudoviricetes* class and is a virulent phage. 

### 2.9. Evolution of Phage 

**Figure 6:**
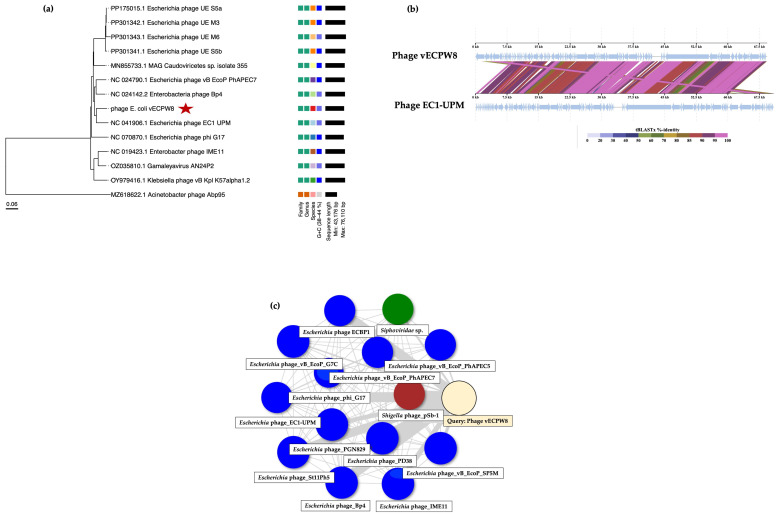
Genetic relationship between *Escherichia* phage vECPW8 and other phages. (**a**) A phylogenic tree of the entire genome of *Escherichia* phage vECPW8, marked with a red star, alongside other phages. This tree was constructed using ViPTree. (**b**) A comparison of the genomes of phage vECPW8 and *Escherichia* phage EC1, created using ViPTree. (**c**) The relationship of phage vECPW8 to other phages in the PhageClouds database.

To investigate the evolution of phage vECPW8 and other related phages, a phylogenetic tree was constructed using the ViPTree web server. The analysis grouped phage vECPW8 as a member of the *Schitoviridae* family, and its host group is the *Pseudomonadota* phylum (Figure 6a). Phage vECPW8 showed a close relationship with *Escherichia* phage EC1-UPM (NC_041906), which was isolated from Malaysia. The results indicate that phage vECPW8 belongs to the *Gamaleyavirus* genus, *Enquatrovirinae* subfamily, *Schitoviridae* family, *Caudoviricetes* order, *Uroviricota* phylum, *Heunggongvirae* kingdom, and *Duplodnaviria* realm. 

A genome-wide alignment between phage vECPW8 and *Escherichia* phage EC1-UPM was performed (Figure 6b). The results revealed variable genome similarity rates between the two phages. While certain regions of their genomes showed high similarity, other regions displayed lower similarity. The novel status of phage vECPW8 was further confirmed using the PhageClounds database (Figure 6c).

### 2.10. The Ability of the Phage to Prevent Biofilm Formation

**Figure 7:**
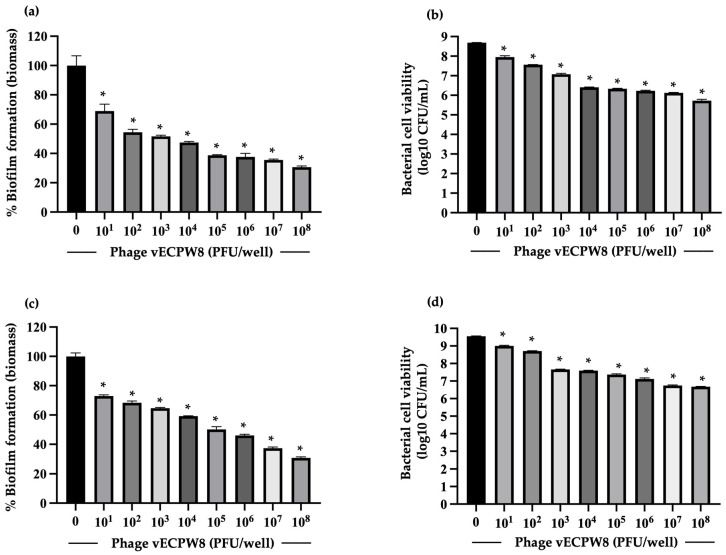
Efficacy of *Escherichia* phage vECPW8 in preventing biofilm formation. APEC host bacteria were treated with different concentrations of phage vECPW8. The biofilm biomass and viable cell counts were measured at (**a**,**b**) day 1 and (**c**,**d**) day 3 post-incubation. Bars represent the standard error of the mean (SEM) and asterisks indicate significant differences (* *p* ≤ 0.05).

The effectiveness of phage vECPW8 to reduce biofilm formation was assessed at 1 and 3 days post-infection by measuring biofilm biomass and colony-forming units (CFUs). For the 24 h biofilms, treatment with phage vECPW8 at concentrations ranging from 10^1^ to 10^8^ PFU/well led to reductions in biofilm biomass of between 31.05 and 69.47% (Figure 7a). Moreover, phage vECPW8 treatment at these concentrations significantly decreased CFU counts by approximately 0.57 to 2.96 log units compared to the untreated control (Figure 7b). For the 3-day-old biofilms, biomass was significantly reduced by between 27.07 and 69.23% after treatment with phage vECPW8 at concentrations ranging from 10^1^ to 10^8^ PFU/well (Figure 7c). The viable bacterial cell count within the biofilm was significantly reduced by 0.37 to 2.88 log units, respectively (Figure 7d). 

### 2.11. The Ability of the Phage to Remove Preformed Biofilms

**Figure 8:**
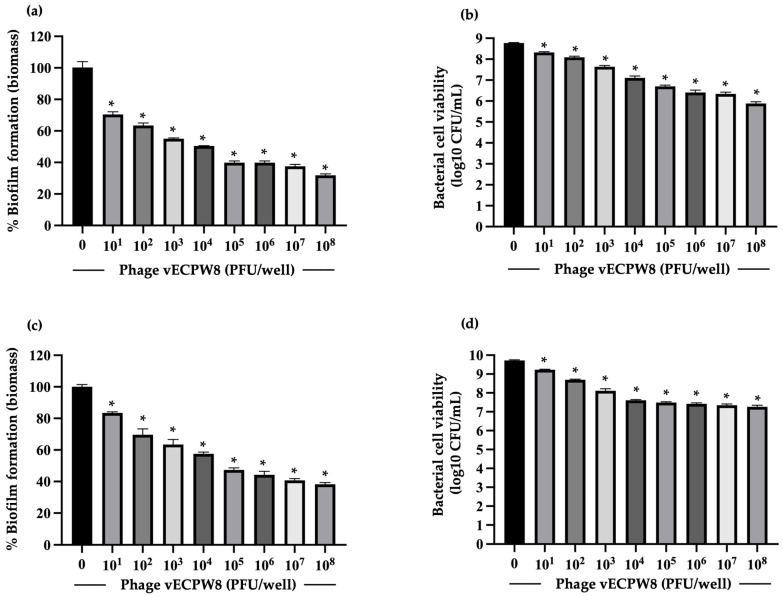
Efficacy of *Escherichia* phage vECPW8 in reducing biofilm formation. Biofilms were allowed to develop for either 1 or 3 days before being treated with various concentrations of phage vECPW8 for 24 h. The biomass and the viable cell counts of (**a**,**b**) the 1-day-old biofilms and (**c**,**d**) the 3-day-old biofilms were measured by crystal violet staining and colony counting, respectively. Bars represent the standard error of the mean (SEM) and asterisks indicate significant differences (* *p* ≤ 0.05).

APEC was allowed to form biofilms for either 1 day or 3 days. At the specified time points, the biofilms were treated with phage vECPW8 at concentrations ranging from 10^1^ to 10^8^ PFU/well for 24 h. For the 1-day-old biofilms, treatment with phage vECPW8 resulted in significant reductions in the total biofilm biomass of approximately 29.51 to 68.15% (Figure 8a). The bacterial viability within these biofilms decreased by approximately 0.38 to 2.83 log units (Figure 8b). For the 3-day-old biofilms, phage vECPW8 at concentrations ranging from 10^1^ to 10^8^ PFU/well reduced the biofilm biomass by 16.57 to 61.75% (Figure 8c) and decreased bacterial viability by 0.35 to 2.34 log units (Figure 8d).

### 2.12. SEM Observation of Biofilms 

**Figure 9:**
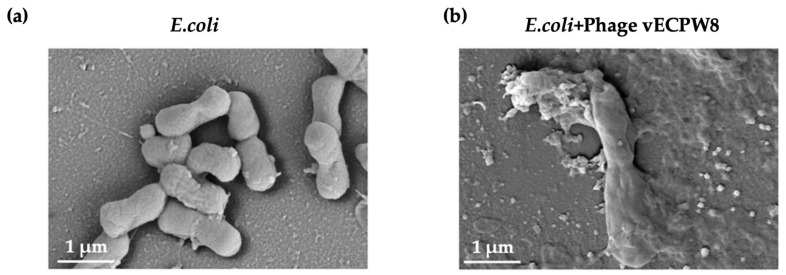
Ultrastructure of biofilms after treatment with *Escherichia* phage vECPW8. Biofilms were grown on glass slides for 3 days and then exposed to phage vECPW8 for 1 day. The biofilms were examined using SEM, comparing (**a**) untreated biofilms and (**b**) biofilms treated with phage vECPW8.

The structure of the biofilms following treatment with phage vECPW8 was evaluated and compared to a control group of biofilms that did not receive phage treatment. Biofilms without phage treatment contained a higher number of bacterial cells than those treated with phage vECPW8 (Figure 9a). In the control biofilms, bacterial morphology appeared normal, whereas biofilms treated with phage vECPW8 exhibited damaged cells (Figure 9b). Evidence of bacterial membrane damage and the presence of holes in the bacterial cell membranes was detected, confirming the antibiofilm activity of phage vECPW8.

## 3. Discussion

APEC infection is a significant concern in broiler farms, as it contributes to severe morbidity and mortality in poultry flocks, leading to a substantial economic burden for farmers. Additionally, this infection is a global concern due to its potential for zoonotic transmission. The possible transfer of antibiotic resistance genes and virulence factors from animals to humans could increase the severity of infections. The use of alternative rational approaches to control APEC with non-antibiotic treatments is gaining attention. Many studies focus on applications of natural products, probiotics, growth inhibitors, antimicrobial peptides, and phage therapy to bacterial controls. In the current study, a phage specific to APEC was isolated, and its biological characterization was included. The antibacterial and antibiofilm efficacies of this phage were assessed. 

Phage vECPW8 was isolated, and electron microscopy revealed that it has a *Podoviridae* morphology. The morphology of the phage under TEM was not optimally clear due to the limitations of the TEM equipment used. However, the classification of the phage was confirmed through whole-genome sequencing, as described later in this study. The host spectrum is a crucial factor affecting the development of phage products. The results showed that phage vECPW8 could lyse many APEC isolates. Adsorption efficacy refers to the ability of a phage to irreversibly adsorb to bacterial cells, which is a crucial factor in determining the timeliness of bacterial eradication [18]. Phages with lower adsorption rates encounter challenges in multiplying [19]. Conversely, if a phage has a higher adsorption rate, bacteria can be killed more quickly during phage therapy [20]. A previous report showed that *Escherichia* phage PEC9, specific to APEC, adsorbed to APEC cells at approximately 84.1% within 10 min, and was characterized as having a high adsorption rate [13]. Similarly, phage vECPW8 adsorbed to cells at a rate of over 85% within 10 min, indicating its high adsorption efficacy. The adsorption rate of a phage to bacterial cells is a crucial factor influencing the effectiveness of bacterial killing [18]. Phages with high adsorption rates rapidly attach to bacterial cells, allowing them to initiate their life cycle sooner, thereby accelerating the phage infection cycle [21]. The growth curve, or infection cycle, of phage vECPW8, an important aspect of its replication, indicated a shorter latent period than that of *Escherichia* phages SKA49 and SKA64, which had latent periods of 35 and 30 min, respectively [14]. This shorter latent period suggests that phage vECPW8 replicates more rapidly, potentially enhancing its effectiveness for phage therapy applications [22]. Moreover, during their life cycles, phages with larger burst sizes are associated with higher rates of phage production. Therefore, phages with a large burst size are preferable due to their ability to produce more progeny after each round of infection. For instance, *Escherichia* phage PEC9 and phage AG- MK-2022 Basu had burst sizes of 68 PFU/mL and 152 PFU/mL, respectively [13,15]. Thus, our results indicated that phage vECPW8 also has a large burst size. The stability of the phage under various environmental conditions, such as different temperatures, pH levels, and UV radiation, is essential for its application. Phage vECPW8, specific to Thai APEC isolates, demonstrated good stability across these conditions, suggesting its potential for development as a phage product.

Whole-genome sequencing revealed a genome length of 69 kilobases. Phage genome annotation using the PHASTEST web server was performed with Prodigal, BLAST+, and Diamond BLAST [23]. In contrast, phage annotation by the PhageScope program was conducted based on data from various public repositories and published databases [24]. Due to the differences in the databases used by PHASTEST and PhageScope, both annotation pipelines were applied in this study. Homology comparison identified that phage vECPW8 shared 65.4% sequence identity with *Escherichia* phage EC1-UPM. Certain regions of the genomes of phage vECPW8 and phage EC1-UPM exhibited high similarity, with over 80% identity, while other regions showed lower similarity (specifically, less than 80%). Notably, the structural protein of phage vECPW8 shared 61.26% identity with that of phage EC1-UPM. This protein is associated with the formation of phage particles, including head and tail structures [25]. Additionally, the tail fiber protein of phage vECPW8 displayed 63.66% identity with that of phage EC1-UPM. Since tail fiber proteins mediate the binding of phages to specific receptors on the bacterial surface, they affect the entry of phages into bacterial cells [26]. Phage tails exhibit high specificity. Therefore, phage vECPW8 and phage EC1-UPM may have varying binding affinities to different *E. coli* isolates, potentially leading to the lysis of distinct bacterial populations. Furthermore, a hypothetical protein found in phage vECPW8 was absent in the genome of phage EC1-UPM. The function of this protein remains unknown, warranting further investigation. Previous studies have reported that a phage with less than 95% identity to any phage in the database would be classified as a new phage [27]. The novelty of phage vECPW8 was confirmed by analysis using the PhageClouds server [28], indicating that phage vECPW8 is a novel phage within the *Gamaleyavirus* genus. 

Phage depolymerases, which are enzymes encoded by phages that specifically degrade the extracellular polysaccharides of bacteria, have proven effective in both preventing and eradicating biofilms in various bacteria [29,30]. For instance, a study on a phage specific to APEC demonstrated that Dpo42, a novel depolymerase derived from *Escherichia* Phage vB_EcoM_ECOO78, exhibited dose-dependent activity in preventing biofilm formation [31]. Many studies have found that phage depolymerases are often located within tail fibers or tail spikes [32,33]. The Phyre2 web server was used to detect a depolymerase domain in the phage genome, which was identified on the tail fiber of phage vECPW8. This finding highlights the potential for investigating the antibiofilm efficacy of phage vECPW8. 

Biofilms are a significant virulence factor of bacteria, including APEC [34]. APEC can form biofilms on both abiotic and biotic surfaces, contributing to their survival and persistence in harsh and nutrient-deficient conditions [35]. Previous studies have also reported that biofilms enhance bacterial resistance to antibiotics and prolong infection. Therefore, controlling biofilms is crucial. Early or young biofilms of some strains can be treated with an antibiotic alone, but mature biofilms are very challenging to manage [36]. Few studies have investigated the antibiofilm activity of phages specific to APEC. For instance, *Escherichia* phage PEC9 could inhibit biofilm formation and reduce bacterial counts in biofilms [13]. A phage cocktail comprising EW2, TB49, G28, AB27, KRA2, and TriM could prevent the biofilm formation of some APEC strains. Moreover, this phage cocktail prevented the proliferation of established biofilms of some APEC strains [37]. Phage vECPW8 was found to inhibit biofilm formation, as evidenced by reduced biofilm biomass and bacterial counts. In preformed biofilms aged 1 day and 3 days, phage vECPW8 reduced both biomass and bacterial counts. The antibiofilm efficacy of phage vECPW8 increased with higher concentrations, showing dose-dependent antibiofilm activity. These results indicated that phage vECPW8 has the potential to prevent biofilm formation and remove preformed biofilms. 

## 4. Materials and Methods

### 4.1. Bacterial Strains and Growth Conditions

Twenty isolates of MDR APEC, maintained as bacterial stocks in our laboratory, were generously provided by Dr. Thotsapol Thomrongsuwannakij from Akkhraratchakumari Veterinary College, Walailak University [38]. The bacterial isolates were resistant to amoxicillin, chloramphenicol, enrofloxacin, nalidixic acid, and tetracycline. The bacterial cultures were grown on tryptic soy agar (TSA; Becton, Dickinson and Company, Franklin Lakes, NJ, USA) for experimental purposes. The bacterial colonies were then transferred into tryptic soy broth (TSB; Becton, Dickinson and Company, Franklin Lakes, NJ, USA) and incubated at 150 rpm for either 6 h or overnight. 

### 4.2. Isolation and Purification of Phage

The phage was isolated and purified using previously established methods [39]. Water samples were collected from Saen Saep Canal in Bangkok, Thailand (latitude = 13°44′57.3966 N; longitude = 100°31′50.754 E), on 15 May 2024. The samples were scooped into sterile falcon tubes and transported to the laboratory in a cool box. The samples were centrifuged at 6000× *g* for 10 min at 4 °C to remove debris. The supernatant was then filtered through a sterile 0.22 μm filter (Merck Millipore, Burlington, MA, USA) to eliminate bacteria. Ten milliliters of the filtrate was mixed with the host MDR APEC PW005 (OD600 = 0.8). The mixture was incubated overnight at 150 rpm and 37 °C. After incubation, the enriched culture was centrifuged at 6000× *g* for 10 min at 4 °C and then filtrated. The diluted host APEC and melted semisolid medium (0.75% agarose) were mixed and poured onto TSA plates. The filtrate was then spotted onto the agar, and the plates were incubated overnight at 37 °C. Plaques were picked and subjected to three rounds of purification using the double-agar overlay method to obtain a purified phage [40].

### 4.3. Large-Scale Amplification of Phage

Phage stocks were prepared using the double-agar overlay method as previously described [39]. Five milliliters of SM buffer was added to the semi-confluent plates, which were then incubated at 4 °C overnight. The supernatant was collected and centrifuged at 6000× *g* for 10 min at 4 °C. The supernatant was filtered through a sterile 0.22 μm filter to obtain the phage stock, which was stored at 4 °C. The phage titer was determined by standard plaque assay and reported as PFU/mL. 

### 4.4. Phage Morphology

Phage morphology was analyzed using TEM through a commercial service provided by the Office of Scientific Instrument and Testing, Prince of Songkla University, Songkhla, Thailand [39]. The phage stock was filtered, and 3 µL of phage suspension was transferred onto a carbon-coated copper grid. The grids were then stained with 2% (*v*/*v*) uranyl acetate (pH 6.7) for 30 s, and excess liquid was blotted off. Phage morphology was observed using a JEM 2010 electron microscope (JEOL, Freising, Germany) operating at 200 kV. Structural details of the phages, such as the heads and tails of the phages, were identified and characterized under TEM. 

### 4.5. Bacterial Lysis Efficiency 

The efficacy of bacterial lysis was determined using the standard spot test method on 20 APEC isolates [40,41]. Exponential-phase bacteria were adjusted to an OD600 of 0.8, and 200 μL of the bacterial suspension was mixed with the melted semisolid medium before being poured onto a TSA plate. Five microliters of the phage, at various dilutions ranging from 10^1^ PFU/mL to 10^8^ PFU/mL, was then placed on the surface and allowed to absorb into the top agar. The plates were incubated overnight at 37 °C, and the lytic zone was detected. The appearance of a lysis zone was reported as (+) for complete lytic or (−) for non-lytic. All experiments were independently conducted in triplicate, and the entire process was repeated twice.

### 4.6. EOP

EOP was performed as previously described [42]. Bacterial isolates that showed susceptibility to the phage were selected for further study using EOP. The phage was serially diluted from 10^1^ PFU/mL to 10^8^ PFU/mL. Subsequently, 200 μL of serially diluted phage solution was mixed with 200 µL of a log-phase bacterial isolate (OD600 = 0.8) and incubated at room temperature for 10 min. The mixtures were then combined with melted semisolid medium and poured onto TSA plates. These plates were incubated overnight at 37 °C. The EOP was calculated as the ratio of the average PFU on target bacteria to the average PFU on host bacteria. EOP values were categorized as follows: high (EOP ≥ 0.5), moderate (0.1 ≤ EOP < 0.5), low (0.001 < EOP < 0.1), or “inefficient” (EOP ≤ 0.001). All experiments were independently conducted in triplicate, with each set including duplicate plaque assays, and the entire process was repeated twice.

### 4.7. Phage Adsorption Rate Assay

The adsorption kinetic of phage vECPW8 was assessed following previously established methods [43,44]. In brief, APEC was incubated with the phage at an MOI of 1. Samples were collected at 1 min intervals for the first 10 min, followed by collection at 5 min intervals from 10 to 20 min post-incubation. After collection, the samples were immediately filtered using a 0.22 μm pore size filter. The filtrate was then diluted and subjected to phage titration using the double-agar layer assay. The adsorption rate constant (*k*), expressed in mL/min, was determined using the formula *k* = (2.3/B*t*) × log(P_0_/P). In the formula, B represents the concentration of bacterial cells (CFU/mL) and *t* is the time interval over which the titer decreases from P_0_ to P. Here, P_0_ and P denote the initial and final concentrations of free virus particles (PFU/mL), respectively. All experiments were independently conducted in triplicate, with each set including duplicate plaque assays, and the entire process was repeated twice.

### 4.8. One-Step Growth Curve 

A one-step growth curve assay was performed as previously described [39,45]. Exponential-phase APEC (OD600 = 0.8) was infected with phage vECPW8 at an MOI of 0.1 and incubated for 10 min at 37 °C to ensure maximum phage adsorption. The mixture was then centrifuged at 6000× *g* for 10 min at 4 °C to remove unabsorbed phages. The pellet was resuspended in pre-warmed TSB and incubated at 37 °C with shaking at 150 rpm. Samples were collected every 10 min over a 120 min period. Phage titers were determined using the double-agar overlay method. The latent period was defined as the time taken by the phage to replicate inside the bacterial cells, while the burst size was calculated as the ratio of the released phage progeny to the initial bacterial cell count. All experiments were independently conducted in triplicate, with each set including duplicate plaque assays, and the entire process was repeated twice.

### 4.9. Inhibition of Planktonic Bacterial Cells by Phage In Vitro

The inhibition of planktonic bacterial cells by phage vECPW8 was evaluated as described previously [39]. Briefly, the host APEC strain was infected with phage vECPW8 at MOIs of 0.01, 0.1, and 1. The samples were incubated at 37 °C with shaking at 150 rpm. Bacterial growth was monitored by measuring the OD at 600 nm using a UV spectrophotometer (Thermo Fisher Scientific Inc., Waltham, MA, USA) every hour for 10 h. After 24 h of incubation, bacterial growth was remeasured. APEC cultures without phage infection served as the control. All experiments were conducted independently in triplicate, and the entire process was repeated twice.

To further evaluate the effect of phage vECPW8 on bacterial cell viability over 24 h, the above methods were repeated. At 24 h post-incubation, samples were collected, diluted in TSB, and plated on TSA plates. The plates were incubated overnight at 37 °C, and the bacterial colonies were counted to determine bacterial cell viability (CFU/mL). All experiments were conducted independently in triplicate, and the entire process was repeated twice.

### 4.10. Visualization of the Phage-Infected Bacterial Cells Under SEM

APEC was infected with phage vECPW8 at an MOI of 0.1, while uninfected APEC served as the control. After incubating at 37 °C for 1 h, the samples were centrifuged at 6000× *g* for 10 min at 4 °C. The supernatant was removed, and the pellets were washed twice with phosphate-buffered saline (PBS). Subsequently, the pellets were resuspended in PBS and deposited onto coverslips. The bacterial cells were then fixed with 2% (*v*/*v*) glutaraldehyde in 0.1 M PBS, followed by incubation at 4 °C for 1 h. After washing twice with PBS, the cells were further fixed with 1% (*v*/*v*) osmium tetroxide (OsO_4_) in deionized (DI) water for 1 h. The coverslips were then washed three times with PBS, and the bacterial cells were dehydrated using a graded ethanol series (20%, 40%, 60%, 80%, and 100%). The coverslips were processed in a critical-point dryer and coated with gold. The bacterial cells were visualized under a field-emission SEM (Zeiss, Oberkochen, Germany).

### 4.11. Effect of Temperature, pH, and UVC Radiation on Phage Stability

For thermal stability testing, phage vECPW8 was incubated at various temperatures: 4 °C, 25 °C, 37 °C, 50 °C, 60 °C, 70 °C, and 80 °C. The phage titer was measured using the double-layer agar method at 1 h post-incubation. For pH stability testing, phage vECPW8 was mixed with SM buffer adjusted to a pH range from 1 to 14, and these samples were incubated at 37 °C for 1 h. The phage titer was then determined using the double-layer agar method. To assess the stability of phage vECPW8 under UVC radiation, the phage was exposed to UVC radiation while positioned on an ice box. The phage suspension was placed in open Petri dishes at a distance of 30 cm from the UVC light source. Samples were collected at 10 min intervals over a 60 min period (0 to 60 min) and then subjected to titer determination. All experiments were independently conducted in triplicate, with each set including duplicate plaque assays, and the entire process was repeated twice.

### 4.12. Whole-Genome Characterization and Analysis

The whole genome of phage vECPW8 was sequenced using the Illumina sequencing platform (Macrogen Inc., Seoul, Republic of Korea). The genomic DNA of phage vECPW8 was extracted, and libraries were prepared using the TruSeq Nano DNA library preparation kit (Illumina, CA, USA). The quality of the library was validated using an Agilent Technologies 2100 Bioanalyzer (Agilent Technologies, Santa Clara, CA, USA) before proceeding using high-throughput sequencing. Data quality was evaluated using FastQC (version 0.11.5), and the filtered reads were assembled into a contig using SPAdes de novo (version 3.15.0). Gene annotation and genome map construction, including predictions for tRNA and tmRNA genes, were performed using the PHASTEST web server (https://www.phastest.ca/submissions/new, accessed on 19 September 2024) [23]. Additionally, the genome was annotated with the PhageScope web server (https://phagescope.deepomics.org/, accessed on 19 September 2024). 

To compare phage vECPW8 with other phages, the VipTree web server was used to construct a phylogenetic tree and perform genome alignments. The EMBOSS Stretcher analysis web server facilitated the analysis of the new phage species by comparing genomic synteny and sequence identity (https://www.ebi.ac.uk/jdispatcher/psa/emboss_stretcher, accessed on 19 September 2024) [46]. The novelty of the phage was assessed with PhageClouds (https://phageclouds.dk, accessed on 19 September 2024) [28]. Additionally, the function of the tail fiber protein was predicted using the PHYRE automatic fold recognition server (https://www.sbg.bio.ic.ac.uk/phyre/html/, accessed on 19 September 2024) [47].

### 4.13. Prevention of Biofilm Formation by the Phage 

The ability of phage vECPW8 to prevent biofilm formation was assessed using a previously published method [39,48]. Phage vECPW8 was diluted tenfold with TSB in flat-bottomed 96-well microtiter plates. A hundred microliters of APEC (OD600 = 0.8) was added into the wells and gently mixed. The final concentrations of phage vECPW8 in the wells ranged from 10^1^ to 10^8^ PFU/well. The plates were incubated without shaking at 37 °C for 1 and 3 days. At these time points, planktonic bacteria cells were removed, and the wells were washed twice with 200 μL of PBS. Biofilm biomass and viable cell counts were evaluated separately in other plates. 

To assess biofilm biomass, 200 μL of 0.1% crystal violet was added to each well, and the plates were incubated for 30 min. The wells were then washed three times with 300 μL of DI water, and the biofilm biomass was solubilized with 200 μL of absolute ethanol. Absorbance was measured at OD595 using a standard microplate reader. The determination of viable cell counts was conducted in parallel. Planktonic bacterial cells were removed, and the wells were washed three times with 200 μL of PBS. Biofilms were resuspended by adding 200 µL of TSB and vigorously pipetting. Serial dilutions of the suspension were plated on TSA plates. The plates were incubated overnight at 37 °C, and bacterial colonies were counted. All experiments were independently conducted in triplicate, and the entire process was repeated twice. The percentage of biofilm formation was calculated using the following formula: ((A_phage treatment_ − A_control_)/A_0_) ×100%, where A_phage treatment_, A_control_, and A_0_ represent the absorbance of the phage-treated, negative control, and untreated control biofilms, respectively [49]. 

### 4.14. Biofilm Eradication of the Phage

The efficacy of phage vECPW8 in removing preformed biofilms was evaluated according to a previous report [11]. A hundred microliters of APEC (OD600 = 0.8) was added to new flat-bottomed 96-well microtiter plates and cultured at 37 °C without shaking for either 1 or 3 days. The media in each well were replaced with 200 μL of fresh media daily. At the specified times, the media were removed, and the wells were washed three times with PBS. Phage vECPW8 was serially diluted tenfold with TSB, and 200 µL of the diluted phage was transferred to the wells. The plates were then incubated at 37 °C for 24 h. Following incubation, the media were removed, and the wells were washed three times with PBS. Biofilm biomass and bacterial cell viability were determined as described above. All experiments were independently conducted in triplicate, and the entire process was repeated twice. The percentage of biofilm was calculated using the formula described above. 

### 4.15. Observation of Bacterial Biofilm Morphology by SEM 

The efficacy of the phage in removing and reducing biofilms was analyzed using SEM. Biofilms were grown on coverslips for 3 days, followed by treatment with phage vECPW8 at a concentration of 10^8^ PFU/mL. One day after treatment, planktonic bacterial cells were removed, and the coverslips were washed three times with PBS. The biofilms were then fixed and dehydrated using a series of alcohol solutions before preparing the samples for SEM, as previously described.

### 4.16. Accession Numbers of the Genome Data

The genome sequence of phage vECPW8 has been deposited in the GenBank database under the accession number PQ362703.

### 4.17. Statistical Analyses

All data were analyzed using GraphPad Prism program, version 10 (GraphPad Software, https://www.graphpad.com/scientific-software/prism/, accessed on 28 October 2024). Statistical significance was assessed using an unpaired *T*-test in GraphPad Prism, with *p* < 0.05 considered statistically significant.

## 5. Conclusions

Phage vECPW8, a novel phage specific to an APEC strain, was isolated. The phage exhibited high lytic activity, a short latent period, good stability under various environmental conditions, and a novel genetic background. It showed promising antibacterial and antibiofilm activities, making it a potential alternative for controlling infections caused by APEC and for use as a biocontrol agent. 

## Figures and Tables

**Table 1:** Bacterial lysis efficacy and EOP of *Escherichia* phage vECPW8.

Strain	Phage vECPW8	
	Lytic Activity	EOP
MDR APEC PW001	-	-
MDR APEC PW002	+	High (0.8)
MDR APEC PW003	+	High (0.65)
MDR APEC PW004	-	-
MDR APEC PW005 *	+	High (Host = 1)
MDR APEC PW006	+	Moderate (0.49)
MDR APEC PW007	-	-
MDR APEC PW008	+	Moderate (0.57)
MDR APEC PW009	-	-
MDR APEC PW010	+	Moderate (0.32)
MDR APEC PW011	-	-
MDR APEC PW012	+	Moderate (0.44)
MDR APEC PW013	+	Low (0.02)
MDR APEC PW014	-	-
MDR APEC PW015	+	Moderate (0.41)
MDR APEC PW016	+	High (0.79)
MDR APEC PW017	-	-
MDR APEC PW018	+	Low (0.03)
MDR APEC PW019	-	-
MDR APEC PW020	-	-

Results were recorded as +, infection, or -, no infection. *, host strain used for the isolation of *Escherichia* phage vECPW8. The EOP values were classified into 4 groups: high production (a ratio ≥ 0.5), moderate production (a ratio 0.1 ≤ EOP < 0.5), low production (a ratio 0.001 < EOP < 0.1) and no production (a ratio ≤ 0.001). Experiments were conducted independently in triplicate, with each set including duplicate plaque assays, and the entire process was repeated twice.

## Data Availability

Data are contained within the article and Supplementary Materials.

## Supplementary Materials

The following are available online at https://www.mdpi.com/article/10.3390/antibiotics13111083/s1, Table S1: Annotation of the specific function of ORFs in phage vECPW8 genome was conducted using PHASTEST. Table S2: Annotation of the specific function of ORFs in phage vECPW8 genome was conducted using PhageScope.
